# Minimally Invasive Resection of Intralobar Pulmonary Sequestration Following Same-Day Interventional Radiological Embolisation of the Feeding Vessel: A Case Series From a Single Tertiary Centre

**DOI:** 10.7759/cureus.106254

**Published:** 2026-04-01

**Authors:** Sarah Fennelly, Kate Nolan, Jyothirmayi Velaga, Cephas Tamburayi Kamba, Bibhusal Thapa

**Affiliations:** 1 Breast Surgery, The Northern Hospital, Epping, AUS; 2 General Surgery, The Northern Hospital, Epping, AUS; 3 Radiology, The Northern Hospital, Epping, AUS; 4 Interventional Radiology, The Northern Hospital, Epping, AUS; 5 Thoracic Surgery, The Northern Hospital, Epping, AUS

**Keywords:** benign lung disease, congenital lung disease, interventional radiology, intralobar sequestration, minimally invasive approach, pre-operative embolisation, pre-operative planning, pulmonary sequestration, radiological anatomy, video-assisted thoracoscopic surgery

## Abstract

Pulmonary sequestration (PS) is a congenital abnormality of the lung where a region of pulmonary parenchymal tissue is supplied by an aberrant systemic artery, which may arise from the thoracic aorta, abdominal aorta, celiac trunk, or intercostal arteries. They are further sub-classified as intralobar or extralobar, depending on whether the sequestered lung has an independent visceral pleural covering. Hybrid minimally invasive treatment of intralobar PS with preoperative embolisation of feeding vessels followed by video-assisted thoracoscopic surgery (VATS) resection has become more widespread in recent years due to increased access to interventional radiology. Embolisation of the feeding vessel is thought to reduce the risk of haemorrhage, which can occur with traction of the delicate feeding vessel wall during VATS.

Here, we present a series of four cases of intralobar sequestration treated by a hybrid minimally invasive approach at our centre from 2021 to 2024. These included three sequestrations with a feeding artery arising from the descending thoracic aorta and one with a feeding vessel arising from the proximal celiac trunk. Each patient underwent interventional radiological (IR) embolisation of the feeding vessel on the morning of surgery, with identification of the vessel by preoperative computed tomography (CT). Two resections were done by a VATS approach, and two others via mini thoracotomy. Ligation of aberrant vessels was done with Endo GIA™ stapler (Medtronic, Minneapolis, MN). The procedure was generally well tolerated, with complications including serous drainage from surgical wounds in two cases. A hybrid minimally invasive approach was implemented successfully in our unit with good outcomes for all four patients. With thorough preoperative planning and cooperation between radiology and surgical units, preoperative embolisation can allow for less invasive thoracic surgery, faster recovery and better outcomes for patients with intralobar PS.

## Introduction

Pulmonary sequestration (PS) describes a congenital lung malformation (CLM) in which an area of pulmonary parenchyma is perfused by an aberrant systemic artery and is not continuous with the tracheobronchial tree [1]. PS represents 0.15-6.4% of all CLMs, with incidence increasing, most likely due to increased frequency of incidental findings [2]. Symptoms of PS may include recurrent pneumonia, haemoptysis, persistent cough or dyspnoea [3-5], but in many cases can be asymptomatic. CLMs arise due to abnormal organogenesis or dysregulation of cellular signalling pathways within the epithelial-mesenchymal interaction during embryonic development [6]. Patients with symptomatic CLMs are usually recommended for surgery. For asymptomatic patients, surgery is also recommended to prevent infection and confirm the diagnosis. It is also recommended because chronic inflammation can increase the risk of malignant transformation over time.

Preoperative embolisation of the aberrant artery allows for a minimally invasive approach while minimising the risk of haemorrhage due to damage to the aberrant artery [7]. A minimally invasive approach is preferable due to reduced postoperative pain, improved quality of life [8], improved recovery time and lower complication rates [9]. The hybrid approach of preoperative embolisation followed by minimally invasive thoracic surgery used in our unit maximises the benefits of both embolisation and video-assisted thoracoscopic surgery (VATS) resection for patients with PS.

## Case presentation

Methods

Here, we present a retrospective review of adult patients aged 30 to 61 years who were treated for PS between 2021 and 2024 at our thoracic surgical centre. Inclusion criteria were any patient over the age of 18 years found to have PS during 2021-2025, with normal lung function and no cardiovascular comorbidities. Exclusion criteria included any patient younger than 18 years, patients with reduced lung function and patients with significant cardiovascular comorbidities. All patients underwent preoperative respiratory function tests, radiological imaging and anaesthetic review. The surgical approaches included mini-thoracotomy and VATS. 

Operative specimens were sent to the laboratory postoperatively for histopathology, microscopy and culture for bacterial growth. Patients were monitored postoperatively in the hospital and re-reviewed in an outpatient setting following discharge. We have outlined the patient demographics, preoperative radiological findings, surgical approach and postoperative outcomes below. 

Results

Four patients were treated for PS in Northern Health between 2021 and 2024. The patient demographics are listed in Table 1. 

**Table 1:** Summary of patient demographics and presenting symptoms

Case	Gender	Age (years)	Smoking status	Presenting symptom
Case 1	Male	30	Never smoker	Persistent shortness of breath post pneumonia
Case 2	Female	44	Smoker	Incidental finding
Case 3	Female	37	Ex-smoker	Upper respiratory tract infection symptoms
Case 4	Male	61	Smoker	Incidental finding

Radiological Findings

**Figure 1:**
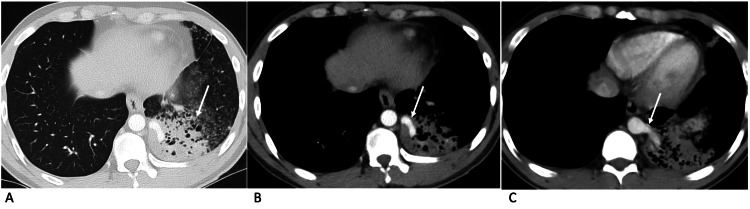
Case 1 - axial images of contrast-enhanced CT of the thorax Axial images of contrast-enhanced CT of the thorax in lung window (A) and mediastinal window (B and C) demonstrate pulmonary sequestration in the left lower lobe (arrow in A) supplied by an arterial branch arising directly from the descending thoracic aorta (arrows in B and C).

**Figure 2:**
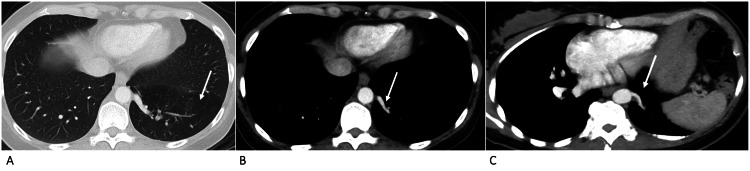
Case 2 - axial images of contrast-enhanced CT of the thorax Axial images of contrast enhanced CT of the thorax in lung window (A), mediastinal window (B), and reformatted maximum intensity projection mediastinal window (C) demonstrates pulmonary sequestration in the posterior segment of the left lower lobe seen as hyperinflation (arrow in A) and supplied by an arterial branch arising directly from descending thoracic aorta (arrows in B and C).

**Figure 3:**
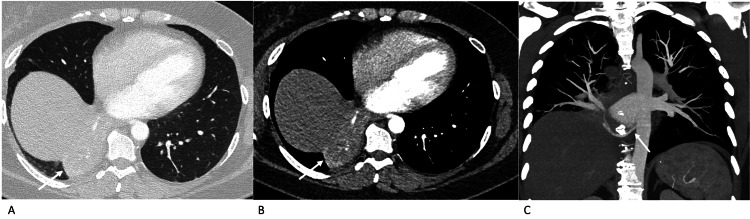
Case 3 - axial contrast-enhanced CT of the thorax Contrast-enhanced CT of the thorax, axial lung window (A), axial mediastinal window (B) and coronal maximum intensity projection (C). Pulmonary sequestration in the right lower lobe is seen as an area of consolidation (arrows in A and B) supplied by an arterial branch arising directly from the descending thoracic aorta (arrow in C).

**Figure 4:**
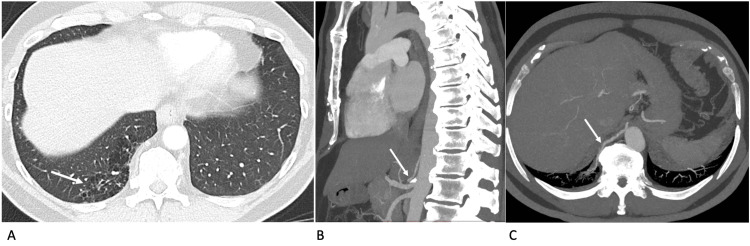
Case 4 - contrast-enhanced axial CT thorax Contrast-enhanced axial CT thorax in lung window (A), coronal mediastinal window maximum intensity projection (B) and axial mediastinal window maximum intensity projection (C). Pulmonary sequestration in the right lower lobe posteromedial segment is seen as an area of multiple cystic segments (arrow in A) supplied by an arterial branch arising directly from the celiac trunk (arrow in B). Note the course of the supplying artery coursing to the sequestration (arrow in C).

**Figure 5:**
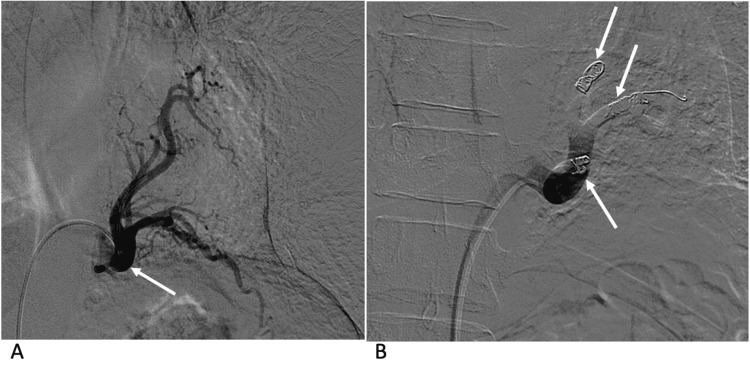
Case 1 - conventional angiography with selective catheterisation of the left lower lobe arterial supply Conventional angiography with selective catheterisation of the left lower lobe arterial supply (arrow) (A). Gelatin sponge (Gelfoam®, Pfizer Inc., New York, NY) was deployed to each of the branches of the major vessel, followed by Nester® coils (Cook Medical, Bloomington, IN) deployment of the branches (arrows) (B).

**Figure 6:**
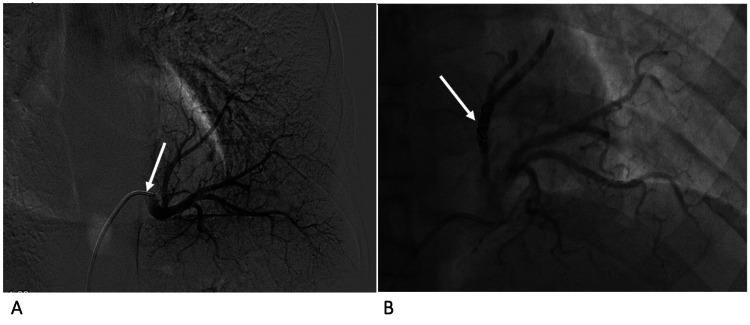
Case 2 - conventional angiography with selective catheterisation of the left lower lobe arterial supply Conventional angiography with selective catheterisation of the left lower lobe arterial supply (arrow in A). Coils (Interlock™ Fibered IDC Occlusion System, Boston Scientific, Marlborough, MA) were deployed in the superior sequestration branch (arrow in B) to aid intraoperative localisation, followed by distal glue embolisation.

**Figure 7:**
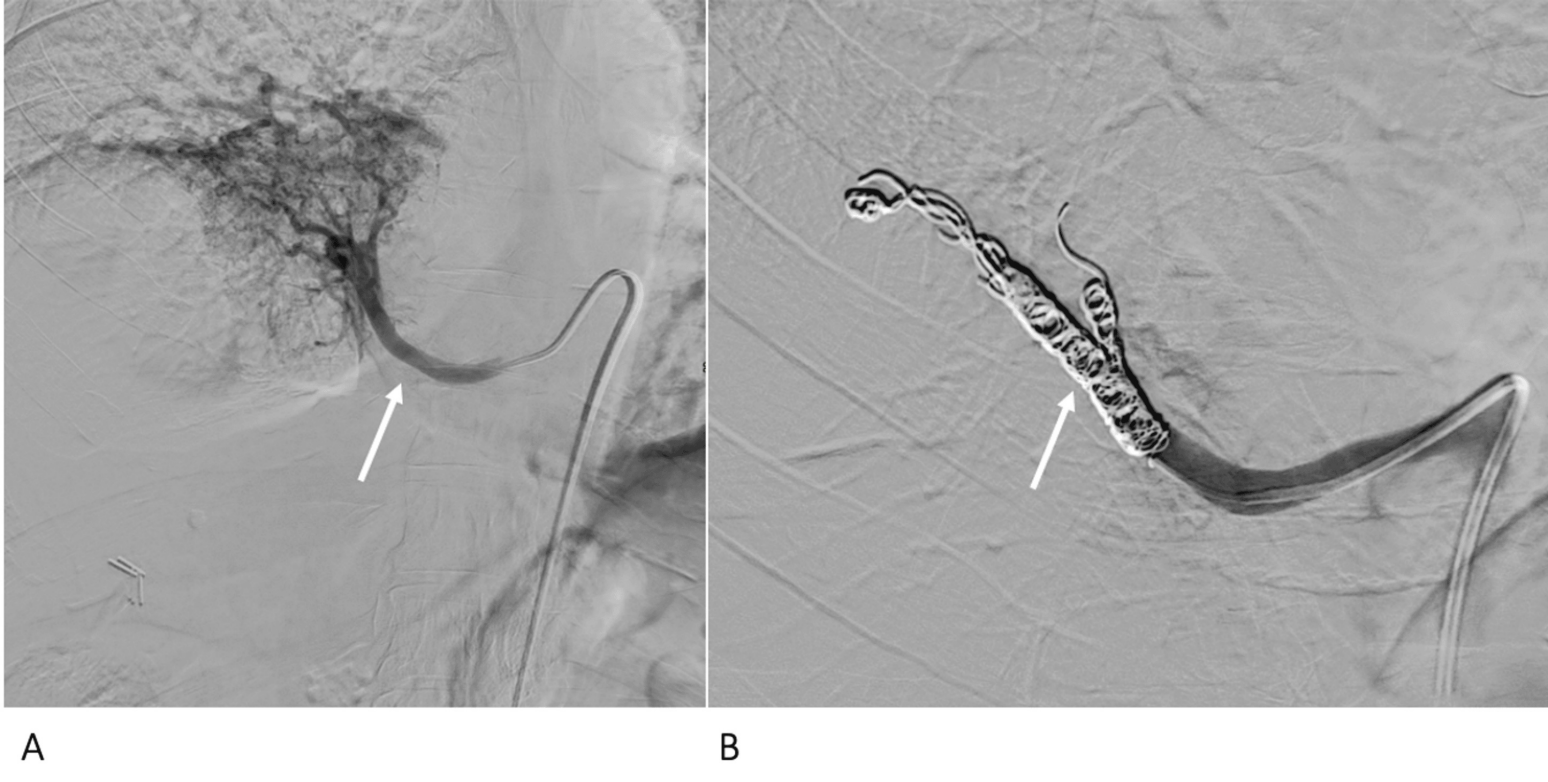
Case 3 - conventional angiography with selective catheterisation of the arterial supply Conventional angiography with selective catheterisation of the arterial supply (arrow) (A) of the right lower lobe sequestration, which was subsequently embolised by coils (arrow) (B).

**Figure 8:**
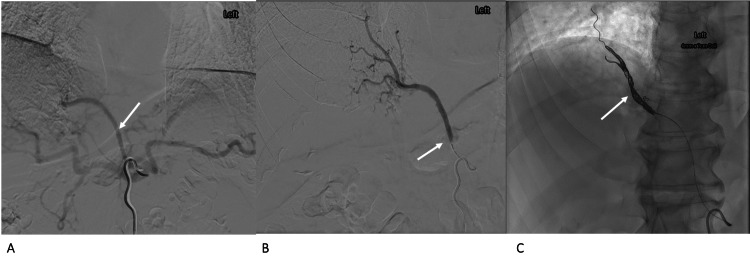
Case 4 - conventional angiography with selective catheterisation of the celiac axis Conventional angiography with selective catheterisation of the celiac axis (A) demonstrates arterial supply to the sequestration from the celiac axis (arrow in A), which was catheterised superselectively (arrow in B). The artery was successfully coiled (C).

**Figure 9:**
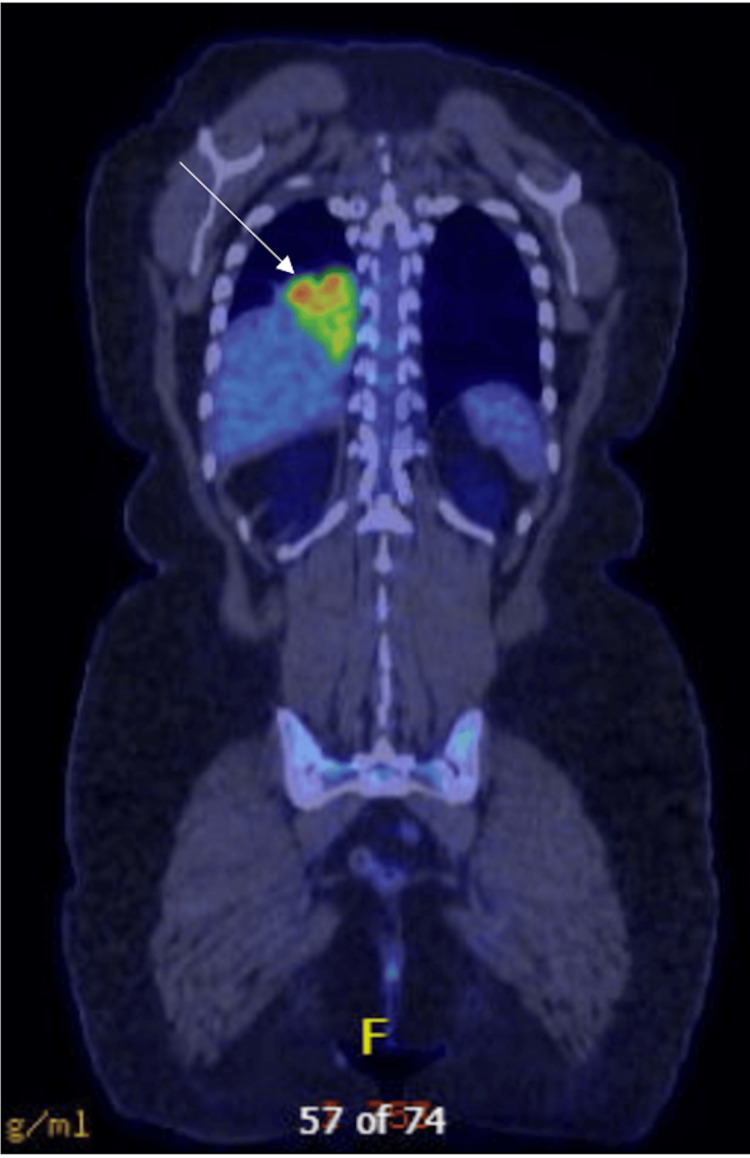
Case 3 - preoperative PET scan Preoperative PET scan demonstrating avidity in the sequestered region of the right lower lobe.

All four patients underwent computed tomography (CT) chest preoperatively (Figures 1-4), demonstrating anatomical variations described in Table 2. CT imaging aided in determining the size and location of the lung involved, and in identifying the aberrant feeding artery to target for embolisation (Figures 5-8). All four CTs confirmed intralobar sequestration, two involving the left lower lobe (Figure 1 and Figure 2) and two involving the posteromedial right lower lobe (Figure 3 and Figure 4). Three out of four of the aberrant arteries supplying the sequestered lung originated from the descending aorta (Figures 1-3, Figures 5-7), and one originated from the proximal coeliac trunk (Figure 4 and Figure 8). In addition to preoperative CT, one patient underwent positron emission tomography (PET) imaging and endobronchial ultrasound (EBUS) to rule out malignancy. PET imaging in this patient reported a moderately fluorodeoxyglucose (FDG) avid lung mass most consistent with reactive adenopathy (Figure 9), and EBUS samples were negative for malignancy.

**Table 2:** Summary of aberrant anatomical findings

Case	Location of pulmonary sequestration	Aberrant arterial vascular supply	Venous drainage of sequestered segment
Case 1	Left lower lobe	Descending thoracic aorta	Left inferior pulmonary vein
Case 2	Left lower lobe	Descending thoracic aorta	Undetermined
Case 3	Right lower lobe	Descending thoracic aorta	Undetermined
Case 4	Right lower lobe	Proximal celiac trunk	Right inferior pulmonary vein

Histopathology and Bacterial Growth

One specimen showed light growth of Nocardia with acute bronchitis and bronchiectasis with extensive lung remodelling, fibrosis and chronic inflammation in keeping with past pneumonia. This patient’s presenting complaint was persistent shortness of breath following an episode of pneumonia.

The patient who had presented with upper respiratory tract infection symptoms initially had some growth of Haemophilus influenzae in the specimen, but given that she was asymptomatic at the time of surgery, this was not treated. Of the two patients who had incidental findings, one histopathology report described normal alveolar architecture, while the other showed some cystic and inflammatory change with no bacterial growth. No specimens contained any malignant or dysplastic foci.

Interventional Radiological Embolisation Approach and Findings

In all four cases, the anatomy of vascular arterial supply was discussed by the interventional radiologist and thoracic surgeon preoperatively. The discussion mainly centred on the surgical clamping site for the feeding artery and the surgical operative approach. The embolisation was planned to be distal to the surgical clamping site. 

All patients had informed written consent for the procedure. Renal function and coagulation profile were checked preoperatively. Transfemoral access was used in all cases, and different appropriate selective catheters were used to access the respective feeding arteries. A coaxial technique using microcatheters was utilised in all four cases. The embolic material used was at the discretion of the operating interventional radiologist. Embolic agents used included pushable and detachable coils, a cyanoacrylate-based liquid embolic agent and absorbable gelatin sponge.

In Case 1, embolisation was performed using a combination of 4-mm pushable fibered Nester® coils (Cook Medical, Bloomington, IN) and a liquid embolic agent. n-butyl-2-cyanoacrylate (NBCA) glue (Glubran® 2, GEM S.r.l., Viareggio, Italy) was mixed with ethiodised oil (Lipiodol®, Guerbet, Villepinte, France) in a 1:3 ratio and delivered under fluoroscopic guidance.

In Case 2, embolisation was performed using a combination of coils and absorbable gelatin sponge (Gelfoam®, Pfizer Inc., New York, NY). A gelatin sponge slurry was prepared by mixing the material with a 1:1 solution of iodinated contrast medium (iohexol; Omnipaque®, GE Healthcare, Chicago, IL) and normal saline to facilitate fluoroscopic visualisation. Coil embolisation was also performed using detachable coils (Interlock™ Fibered IDC Occlusion System, Boston Scientific, Marlborough, MA), with two 4-mm coils deployed in each of the larger superior branches and two 2-mm coils deployed in each of the smaller inferior branches.

In Case 3, embolisation was performed using coil embolisation alone, including Prestige® peripheral embolisation coils (Balt, Montmercy, France) and Nester® pushable coils, deployed sequentially until angiographic occlusion was achieved.

Similarly, in Case 4, embolisation was performed using coil embolisation alone, with a combination of Prestige® and Nester® coils of varying diameters and lengths selected according to vessel size.

Technical success was defined as angiographic cessation of flow within the target vessel at the completion of the procedure.

There was no reported difference in the degree of devascularisation between cases by the surgical team. The feedback from the thoracic surgeons was that excellent devascularisation was achieved in all four cases.

Surgical Approach and Operative Findings

The VATS procedures were performed under single lung ventilation in a full lateral position. First, the aberrant artery was dissected, closed and cut with a Multifire Endo GIA™ 30 stapler (Medtronic, Minneapolis, MN). Afterwards, a standard thoracoscopic lobectomy, or a resection of the extrapulmonary sequestration, was performed. The first two patients underwent thoracotomy and anatomical left lower lobectomy with an Endo GIA™ stapler. One of these had stapler ligation of the aberrant feeding vessel prior to lobectomy. 

The third and fourth patients had VATS resections, with one having a VATS right lower lobectomy and the other having a division of an aberrant vessel and excision of the sequestered posterior basal segment.

Postoperative Outcomes 

The average operative time for all four cases was three hours. There were no surgical complications. All patients had intercostal catheters inserted intraoperatively, which remained in for one to four days. Two patients experienced fluid discharge from the catheter site following catheter removal, which settled with conservative measures within seven days. One patient was discharged on day 3 postoperatively and re-presented three days later with ongoing fluid discharge, but this also improved with conservative management.

All patients were monitored postoperatively in the intensive care unit and discharged to the ward on day 1 postoperatively. The average length of hospital stay was three days. All patients underwent routine outpatient follow-up two weeks after discharge. All patients reported resolution of their symptoms two weeks post-discharge, and chest radiographs at this time revealed no pneumothorax or haemothorax. No patients developed long-term complications within six months of surgery.

## Discussion

With increased access to CT and other diagnostic imaging, diagnosis rates of asymptomatic PS are likely to increase. Given the rarity of this condition, standardized protocols should be developed for its management based on the cumulative experience of many thoracic surgery units. Here we present our outcomes from the first two years of treating PS by a hybrid approach with the goal of achieving the most minimally invasive intervention possible. It has been suggested in the literature that embolisation of the feeding vessel alone is sufficient to treat PS [10,11]. Other centres proceed directly to resection of the sequestered segment in patients with respiratory problems, recurrent infections, or whose imaging reveals a lesion taking up more than 20% of the hemithorax [12]. 

The main concern with proceeding directly to surgery is the risk of damage to the fragile aberrant feeding vessel, causing major haemorrhage. In our centre, the first two pulmonary sequestration cases were done by thoracotomy to mitigate this risk. However, using a hybrid approach with pre-operative embolisation dramatically reduces this risk. 

Another approach to reduce risk is by pre-operative assessment of the aberrant vessel by multidetector computed tomography (MDCT), but in 2 of the published cases of VATS resection of pulmonary sequestration, MDCT did not reveal several smaller, more thin-walled vessels [13]. 

## Conclusions

A hybrid approach involves the utilisation of more resources in the radiology and surgical departments. However, given the rarity of this condition and the significant risk reduction that is likely conferred by pre-operative embolisation, this approach is reasonable. Future studies will be needed to demonstrate this risk reduction on a statistically significant scale.
